# O064: Validation and assessment of the new surveillance paradigm for ventilator-associated events

**DOI:** 10.1186/2047-2994-2-S1-O64

**Published:** 2013-06-20

**Authors:** MS Van Mourik, PM Klein Klouwenberg, DS Ong, MJ Schultz, J Horn, OL Cremer, MJ Bonten

**Affiliations:** 1 Department of Medical Microbiology, University Medical Center Utrecht, Utrecht,The Netherlands; 2 Department of Intensive Care, University Medical Center Utrecht, Utrecht, The Netherlands; 3 Julius Center for Health Sciences and Primary Care, University Medical Center Utrecht, Utrecht, The Netherlands; 4 Department of Intensive Care Medicine, Academic Medical Center, University of Amsterdam, Amsterdam, The Netherlands

## Introduction

Reliable, meaningful surveillance methods are essential for benchmarking of healthcare-associated infection rates. However case-definitions for ventilator-associated pneumonia (VAP) are complex and subjective. A novel surveillance paradigm for detection of ventilator-associated events (VAE) was recently proposed by the National Healthcare Safety Network (NHSN).

## Objectives

We aimed to validate this new algorithm.

## Methods

Retrospective analysis of an ICU cohort with ongoing prospective assessment of VAP in 2 academic medical centers (January 2011 – June 2012). The VAE algorithm was electronically implemented as specified by NHSN and includes assessment of (infection-related) ventilator-associated conditions (VAC, IVAC) and possible or probable VAP. Incidence and concordance of VAE with prospective VAP surveillance was assessed as were alternative clinical conditions occurring with VAE signals. The attributable mortality of VAC, IVAC and VAE VAP was assessed by competing-risk survival analysis.

## Results

2080 patients contributed 2296 episodes of mechanical ventilation (MV). Incidence of VAC and IVAC were 10.0 and 4.2/1000 ventilation days, respectively. VAP according to the VAE algorithm occurred in 3.2/1000 MV days, whereas prospective surveillance identified 8 cases per 1000 MV days. VAC detected 32% (38/115) of the patients affected by VAP, positive predictive value was 25% (38/152). The other VAE events had lower sensitivity and positive predictive value remained < 40%. VAC signals were most often caused by pulmonary or extra-pulmonary infection, volume overload or heart failure. The subdistribution hazard for mortality was estimated at 3.3 (95% CI 2.4 - 4.4) for VAC, 2.5 (1.5 - 4.1) for IVAC and 2.1 for VAP (1.2 - 3.8).

## Conclusion

The VAE algorithm aims to assess complications of mechanical ventilation. However, concordance between VAE and VAP surveillance is poor. Future studies will need to assess whether the conditions identified as VAE are liable to preventive measures.

## Disclosure of interest

None declared.

